# Clinical Benefit of Niraparib to TKI/mTORi-Resistance Metastatic ccRCC With *BAP1*-Frame Shift Mutation: Case Report and Literature Review

**DOI:** 10.3389/fonc.2022.927250

**Published:** 2022-07-06

**Authors:** Bi-Jun Lian, Ke Zhang, Xu-Dong Fang, Feng Li, Zhao Dai, Wei-Ying Chen, Xiao-Ping Qi

**Affiliations:** ^1^ Department of Oncologic and Urologic Surgery, The 903rd People's Liberation Army (PLA) Hospital, Wenzhou Medical University, Hangzhou, China; ^2^ Center for Radiation Oncology, Affiliated Hangzhou Cancer Hospital, Zhejiang University School of Medicine, Hangzhou, China; ^3^ Department of Neurology, The 903rd People's Liberation Army (PLA) Hospital, Wenzhou Medical University, Hangzhou, China; ^4^ Department of Urology, Taizhou Hospital of Zhejiang Province Affiliated to Wenzhou Medical University, Enze Hospital, Taizhou Enze Medical Center (Group), Taizhou, China

**Keywords:** clear cell renal cell carcinoma, resistance, BAP1 mutation, niraparib, case report

## Abstract

Clear cell renal cell carcinoma (ccRCC) is the most common subtype of renal cancer. The top four mutant genes affecting the occurrence and progression of ccRCC are *VHL, PBRM1, BAP1*, and *SETD2*, respectively. Tyrosine kinase/mammalian target of rapamycin inhibitors (TKI/mTORis) with or without immunotherapy are the standard and effective therapy to metastatic ccRCC. Once TKI/mTORis fail to ccRCC, there is still a lack of other effective therapies. In this study, we reported a case in which a metastatic ccRCC patient (T2aN1M1) presented resistance after a 28-month treatment by sorafenib–axitinib–everolimus (TKI-TKI-mTORi). Subsequently, a frame shift pathogenic mutation, c.799_800del (p.Q267fs) in the exon10 of *BAP1* in ccRCC, was revealed by targeted sequencing. Oral administration of nilapanib (PARP inhibitor) was further given, which may provide a new therapy for TKI/mTORi-resistance metastatic ccRCC. Fortunately, a partial response has been achieved and lasted for 5 months. Since the frequency of *BAP1* mutations in ccRCC patients was approximately 10%–20%, as reported previously, we also tried to explore the potential mechanisms benefitting from the nilapanib. Moreover, the literature concerning *BAP1* mutation and associated cancers including ccRCC is reviewed.

## Introduction

Clear cell renal cell carcinoma (ccRCC) is the most common pathological subtype of renal cell cancer, with a proportion of more than 75%. CcRCC is characterized by a high frequency, more than 90%, of von Hippel–Lindau (*VHL*) gene inactivation, which plays a key role in regulating angiogenesis through a hypoxia-driven pathway and affects the expression of multiple genes, such as the vascular endothelial growth factor (*VEGF*) and its receptor (*VEGFR*) (1–3). Due to the lack of effective chemotherapy, interferon, and cytokines, the tyrosine kinase inhibitor (TKI), which target those pathways including *VEGF* and *VEGFR* genes, played an important role in the treatment of advanced ccRCC in the past 20 years (4). With the development of immunotherapy, especially since 2019, TKI alone is instead by the TKI combined with immunotherapy for the metastatic ccRCC as the first-line therapy (5, 6). Alhough TKI with or without immunotherapy did prolong metastatic ccRCC patients’ progression-free survival (PFS) time. The second line therapy is limited to change one TKI to another (TKI to TKI) or the mammalian target of rapamycin inhibitor (mTORi), and in the absence of precision medicine based on gene sequencing.

Actually, *VHL* is not the only inactivation gene that appeared in ccRCC. Secondarily mutated genes, including *PBRM1, BAP1*, and *SETD2*, are also involved in the formation of ccRCC, with a frequency up to 30%–41%, 10%–20%, and 10%–20%, respectively (7–9). Here, we present a metastatic ccRCC patient with a somatic mutation *BAP1* detected by targeted genes and next-generation sequencing (targeted sequencing; range 808 genes^®^). After a 28-month treatment of TKI to TKI to mTORi, the patient presented resistance and tumor progression based on RECIST v1.1 (10). Subsequently, nilapanib [poly ADP-ribose polymerase inhibitor (PARPi)] was further given, and partial response was achieved, which has not been reported before. This may provide a new insight and drug therapy for advanced-resistance ccRCC. Moreover, we also systematically reviewed the previous literature, and assessed the *BAP1* alteration frequency and possible mechanism in cancers, particularly in ccRCC.

## Case Description

**Figure 1:**
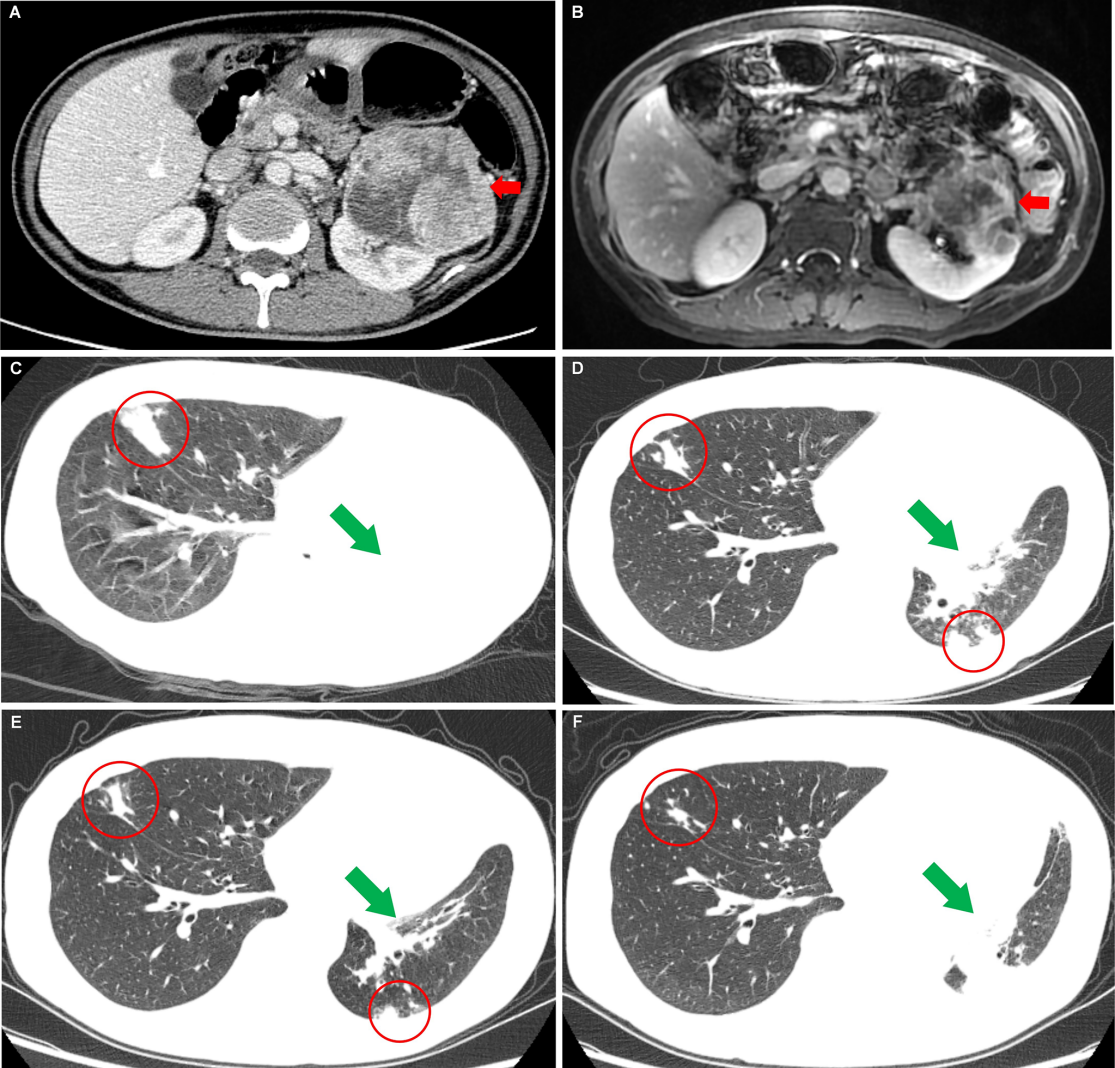
Images of the left renal carcinoma and the changes of metastases when the patient took nilapanib. **(A)** Computed tomography image examination showed an enhancing mass measuring 9.3 cm × 8.2 cm × 7.7 cm (red arrow) in the left renal. **(B)** Magnetic resonance image examination revealed a significantly reduced mass [7.3 cm × 6.8 cm × 7.0 cm (red arrow)] in the left renal after sorafenib-targeted therapy for 14 months. **(C)** Before nilapanib; **(D)** After nilapanib for 2 months; **(E)** After nilapanib for 4 months; **(F)** After nilapanib for 5 months. Chest CT showed metastases in the lungs (red circle) and atelectasis in the left lung (green arrows) due to the tumor invasion of the left bronchus, received continuous remission in the first 4 months after nilapanib **(C–E)**. **(F)** showed that new metastases appeared in the hilus of the left lung, resulting in atelectasis.

A 49-year-old man without urologic symptoms and renal cancer family history received a conventional physical examination in a local hospital in May 2018. A huge hypoechoic mass in the left renal was occasionally showed by urologic Doppler ultrasound (US) examination. Computed tomography (CT) revealed an enhancing mass measuring 9.3 × 8.2 × 7.7 cm in the left renal (**Figure 1A**), multiple enlarged lymph nodes in the retroperitoneal, and a quasi-circular mass (maximum size 3.0 cm) in the liver but normal alpha-fetoprotein levels (1.54 ng/ml; normal <5.0 ng/ml). Afterwards, a US-guided fine-needle aspiration (FNA) was performed on the left renal mass. The histological Hematoxylin-Eosin (H&E) examination of biopsy specimens showed features positive for malignancy, and immunohistochemistry revealed that the tumor cells were positive expressions of PAX-8, CAIX, CD10, and negative for CK7, suggesting ccRCC [T_2a_N_1_M_1_ (11)]. Meanwhile blood routine testing showed the patient with a lower serum hemoglobin of 97 g/L, an elevated platelet of 393 × 10^9^/L, and Ca^2+^ 3.05 mmol/L, respectively. Based on these blood markers and less than 1 year from diagnosis to treatment, the patient was classified as a poor-risk group according to the International Metastatic Renal Cell Carcinoma Database Consortium (IMDC) criteria (11). Sorafenib (400 mg, bid) was subsequently taken as the first-line therapy based on the European Association of Urology (EAU) Guidelines–2018 (11). In July 2019, after the patient achieved a partial response confirmed by magnetic resonance imaging (MRI) after sorafenib-targeted therapy for 14 months (**Figure 1B**) (10), cytoreductive nephrectomy (CN) was further performed. Histopathological examination revealed ccRCC with lymph node metastases. Two weeks later, a radiofrequency ablation of liver metastases was also conducted. After 2 months of the CN operation, sorafenib was replaced by axitinib (5 mg, bid) due to an adverse event of grade 3 maculopapular erythroderma, and the disease was stable continuously. In May 2020, the patient began to lose weight and have a headache. Chest CT identified the areas of new abnormal metabolism in both lungs and brain MRI showed a mass in the right optic canal. Everolimus (10 mg per day; mTORi) was subsequently given as the third line but showed ineffective treatment.

**Figure 2:**
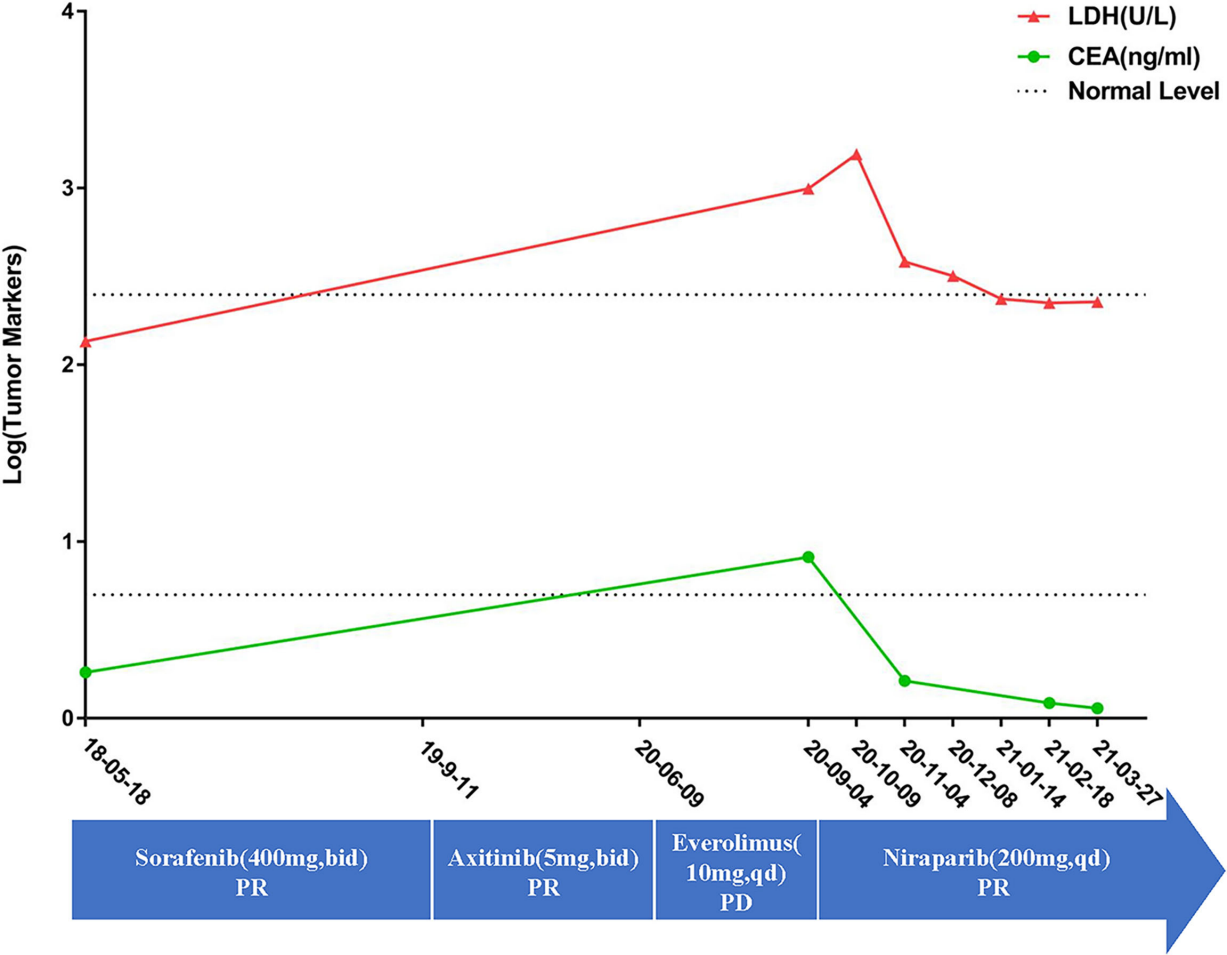
The patient’s detailed treatment process and the tumor marker changes. The curve showed that the tumor markers is still in the normal level upon first diagnosis. However, they were already higher than normal when the patient was admitted in our hospital. However, after taking nilapanib, the tumor markers returned to normal again. The blue arrow showed the detailed medication information along the treatment. On the other hand, there is still the lack of specific tumor markers for ccRCC. The changes of LDH and CEA may be inconsistent with changes in the tumors.

In September 2020, the patient came to our hospital with a severe headache and dyspnea. The admission assessment showed a 4-grade Eastern Cooperative Oncology Group (12) and an 8-grade Visual Analogue Scale (VAS) Pain (13). Chest CT showed atelectasis in the left lung due to the tumor invasion of the left bronchus and metastases in the right lung (**Figure 1C**). Brain MRI showed a mass in the right optic canal. Targeted genetic testing was further performed on a ccRCC specimen from nephrectomy [formalin-fixed paraffin-embedded (FFPE)] by utilizing the Illumina HiSeq 4000 platform. A frame shift pathogenic mutation c.799_800del (p.Q267fs) in the exon 10 of *BAP1* (https://www.ncbi.nlm.nih.gov/clinvar/variation/1070749/) was found. Owing to the patient’s worse performance status and high cost of drugs, immune checkpoint inhibitors (PD-1/PD-L1/CTLA-4/LAG-3/CD47) were not a priority treatment option and should be refused. On the contrary, based on *BAP1* mutation detected in the tumor in the patient, PARPi should be recommended. In October 2020, niraparib (200 mg per day) was taken after obtaining the patient and his family’s full informed consent, followed by CyberKnife radiosurgery (25 Gy; 5 cycles) for the treatment of intracranial metastatic lesion and supplemented nutritional support therapy. To our excited, the patient displayed a partial response in both lungs after niraparib for 2 months (**Figure 1D**). The intracranial lesion also shrunk due to radiotherapy and the headache were totally released. The Lactate Dehydrogenase (LDH) and Carcino-Embryonic Antigen (CEA), which elevated to abnormal when the patient admitted to our hospital, returned to normal again (**Figure 2**). In the following 2 months, the patient received continuous remission (**Figure 1E**). However, in March 2021, 5 months after taking niraparib, new metastases appeared in the brain and the hilus of the left lung, resulting in atelectasis (**Figure 1F**). Then, the patient began to suffer from breathing difficulties and refused any further treatment, except palliative care. Eventually, the patient died in June 2021 according to his family’s message.

**Figure 3:**
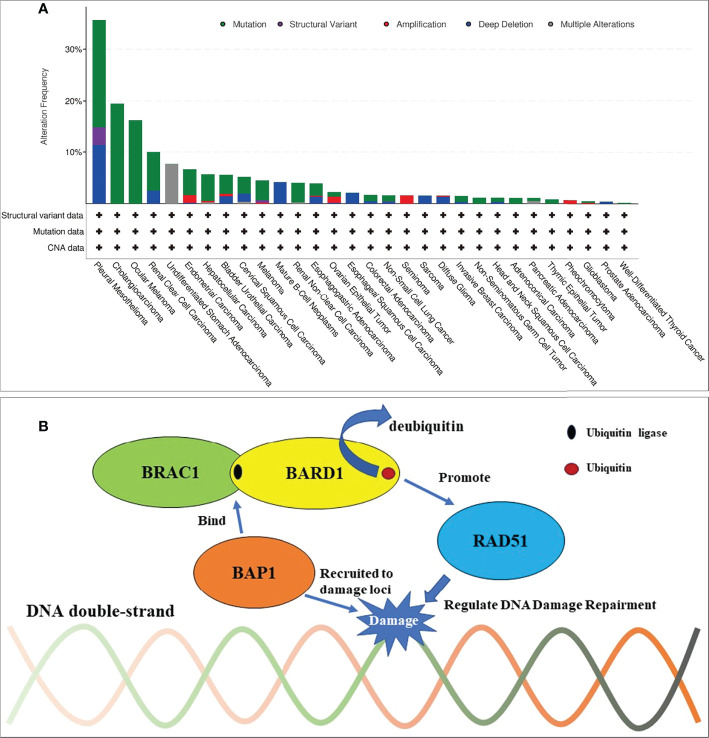
Frequencies of BAP1 alteration across cancer types and mechanism of BAP1 in DNA damage repairment. **(A)** *BAP* mutations most commonly occurred in pleural mesotheliomas, cholangiocarcinoma, ocular melanoma, and renal clear cell carcinoma from a total of 32 large-scale TCGA PanCancer Atlas Studies that contained 10,967 samples. **(B)** Mechanism of BAP1 in DNA damage repairment. BRCA1/BARD1 complex is an E3 ubiquitin ligase and interacts with the recombinase RAD51 to regulate the homologous recombination repairment. BAP1 can be recruited to DNA damage loci and bind to the BRCA1/BARD1 complex. By this, BAP1 can inhibit the E3 ligase activity of BRCA1/BARD1 to protect against additional ubiquitination and deubiquitinate preexisting ubiquitin chains (bent arrow).

Moreover, we clustered data that were identified from other patients with cancer and *BAP1* alteration by the “cBioPortal” (http://www.cbioportal.org). This identified a total of 32 large-scale TCGA PanCancer Atlas Studies containing 10,967 samples. The data from these publications are clustered for analysis as shown in **Figure 3A**.

## Discussion

*BAP1*, which is located on the chromosome 3p21.1, is a ubiquitin carboxy-terminal hydrolase (14). It was recognized as a tumor suppressor, and a mass of processes including chromatin modification, programmed cell death, cell cycle control, DNA damage repair, and the immune response were regulated by its deubiquitinating activity (14, 15). *BAP1* can undergo germline or somatic alterations, and tumors that are relevant to germline are semblable to those with somatic *BAP1* alterations (15). The inactivation of the *BAP1* gene (either germline or somatic form) often led to the development of a number of cancers, such as melanoma, malignant mesothelioma, and ccRCC (14, 16). This may indicate the common mechanisms of tumorigenesis and the potential target therapies to *BAP1* in these highly relevant tumors (15). CcRCC with a *BAP1* mutation often showed clinicopathologic features as a high pathological stage, high renal vein involvement rate, and high metastasis rate and always had a worse prognosis, even in patients with low-risk tumors (17–19). In this study, the presence of the patient by physical examination showed that the occasional ccRCC had advanced and distant (bone and liver) metastasis (T2aN1M1), implying a worse prognosis. Moreover, the traditional targeted therapy for ccRCC with *BAP1* mutation, for example, antiangiogenic therapy-TKI, might also show a poor outcome (20). In contrast, this patient received a 24-month PFS time in total by the two TKI (sorafenib to axitinib) therapies, showing a certain degree of efficacy. On the contrary, mTORi (everolimus) was used but not effective, lasting 3 months continuously. The treatment options for ccRCC patients regardless with or without the *BAP1* alterations are limited to traditional therapies. TKI with or without immunotherapy was still the main treatment method. Once TKI failed, the optimal further treatment is scanty. Immunotherapy was an option for a subsequent treatment; however, patients’ poor performance status and the high cost of immune checkpoint inhibitors always prevented its application, such as this patient. It is interesting that several therapies targeting the *BAP1* alterations showed a positive potential in cancers, according to the recent studies (21, 22). The enhancer of zeste homolog 2 inhibitor (EZH2i) and histone deacetylase inhibitors (HDACis), which target the mechanism of BAP1 in transcriptional regulation and chromatin modification, respectively, are potential targeted therapies (21, 22). An EZH2i study had positive results obtained in a phase 2 trial conducted in *BAP1*-altered malignant pleural mesothelioma *in vivo* (22), as well as HDACi warrants further exploration whether *BAP1* aberrations modulate response in VANTAGE 014 study (21), although BAP1 downregulation increases the sensitivity to HDACi *in vitro* (23). Unfortunately, EZH2i and HDACi were not available in China, potentially, PARPi should be chosen as the further treatment for this TKI/mTORi-resistance advanced ccRCC.

*BAP1* is first known as a protein interacted with *BRCA1*, which is the famous homologous recombination repair gene (24, 25). Nowadays, research has also reported that BAP1 regulates the DNA damage repair in many ways, for instance, binding the BRCA1/BARD1 complex. The BRAC1/BARD1 complex, which functions as an E3 ubiquitin ligase, is known to play a significant role in the DNA damage response through recruiting RAD51 to the damaged loci and so on (26, 27). Jensen et al. showed that BAP1 can interact with BRCA1 and augment the tumor suppressor activity of BRCA1 (28). Nishikawa et al. showed that BARD1 is the major binding partner of BAP1 (29). By binding to the BRCA1/BARD1 complex, BAP1 can inhibit the E3 ligase activity of BRCA1/BARD1 to protect against additional ubiquitination and deubiquitinate preexisting ubiquitin chains (29). The dual role of BAP1 toward BRCA1/BARD1 could be important to ensure the inhibition of ubiquitination in cellular pathways, though the interacting mechanism of these molecules between BAP1 and BRCA1/BARD1 needs further study (**Figure 3B**) (24–30). Thus, PARPi would have a synthetic lethal effect on *BAP1*-mutated tumors, theoretically. Given the known experiment of *BAP1*-mutated cancers, PARPi was effective in the treatment of malignant neoplasm—pleural mesotheliomas, osteosarcomas *in vitro* (31–34) and mesotheliomas, intrahepatic cholangiocarcinoma *in vivo* (35–38), and so on (**Table 1**). The ongoing phase II clinical studies of niraparib and olaparib also react to the safety and feasibility of PARPi in treating patients with *BAP1* alterations (NCT03207347, NCT03531840, NCT03786796). As long as the mutated genes are identical, the same targeted drugs are feasible for different diseases (39). To control tumor progression, PARPi may have the potential to target *BAP1*-altered ccRCC. Based on these findings, niraparib was taken after obtaining the patient’s full informed consent. The exciting part is that the tumors in the lungs achieved a partial response for 5 months, suggesting that niraparib is a relatively effective treatment for TKI-refractory metastatic ccRCC with *BAP1* alteration. Although owing the lack of specific tumor markers for ccRCC, the effect of the treatment cannot always be reflected by tumor markers. The reduction of LDH and CEA, which elevated to abnormal when the patient was admitted to hospital, may indicate that niraparib worked to some extent. Moreover, for ccRCC patients with a *BAP1* alteration, immunotherapy and/or immunocombination therapy may improve efficacy. Recent reports revealed that *BAP1* alterations in cancer confer distinct immunogenic phenotypes that may be particularly susceptible to novel cancer immunotherapies (40). *BAP1* mutations in ccRCC correlate with increased CCR5 expression and immunosuppression (41). These studies speculated that both PARPi and immunotherapy seemed to show enormous potential in treating ccRCC with *BAP1* alteration. Unfortunately, the patient never received the immunotherapy from the beginning to the end.

**Table 1:** Studies of PARPi in tumors with BAP1 alteration.

Tumor phenotype	PARPis	Study type	Comments
Malignant mesotheliomas	Olaparib, niraparib	*In vitro*	PARPis at clinically relevant concentrations result in significant cytotoxicity in malignant mesotheliomas (31).
Osteosarcomas	Talazoparib	*In vitro*	Osteosarcoma cells with genetic signatures of BRCAness are susceptible to the PARPi talazoparib (32).
Malignant mesothelioma	Olaparib	*In vitro*	The response to PARPi could be demonstrated in the BAP1-mutated NCI-H2452 cells (33).
Malignant mesothelioma	Olaparib	*In vitro*	Patients with a high expression of BAP1 may be responsive to PARPi (34).
Malignant mesotheliomas	Olaparib	Phase 2 clinical trial (n=23)	Olaparib is safe with no new safety concerns and has limited activity in previously treated mesothelioma (35).
Malignant mesotheliomas	PARPi	Retrospective cohort study (n=4)	No responses were observed with PARPi (36).
Malignant mesotheliomas	Rucaparib	Phase 2 clinical trail	Rucaparib in patients with BAP1-negative or BRCA1-negative mesothelioma met the prespecified criteria for success (37).
Intrahepatic cholangiocarcinoma	Olaparib	Case report	Following 11.0 months on olaparib treatment, sustained stable disease control is ongoing (38).

PARPi, poly ADP-ribose polymerase inhibitor.

## Conclusion

This study demonstrates that PARPi may be another potential therapy for TKI**/**mTORi**-**resistance ccRCC with a *BAP1* mutation, and, additionally, immunotherapy and/or immunocombination may also have effect on ccRCC with a *BAP1* mutation, although this warrants further validation in clinical studies. An individualized comprehensive approach for advanced ccRCC with *BAP1* mutation is beneficial.

## Data Availability Statement

The original contributions presented in the study are included in the article/**Supplementary Material**. Further inquiries can be directed to the corresponding authors.

## Ethics Statement

The studies involving human participants were reviewed and approved by The 903rd Hospital Ethics Committee. The patients/participants provided their written informed consent to participate in this study. Written informed consent was obtained from the individual(s) for the publication of any potentially identifiable images or data included in this article.

## Author Contributions

Conception/design: X-PQ and B-JL; Provision of study material or patients: KZ, W-YC, FL, and X-DF; Data collection and analysis: B-JL, KZ, W-YC, X-DF, and ZD; Manuscript writing: B-JL and X-PQ; All authors have read and approved the manuscript.

## Funding

This work was supported by the National Natural Science Foundation of China (81472861), the Key Project of Zhejiang Province Science and Technology Plan, China (2014C03048-1), and Hangzhou Municipal Commission of Health and Family Planning Science and Technology Program (B20210355, OO20190253).

## Conflict of Interest

The authors declare that the research was conducted in the absence of any commercial or financial relationships that could be construed as a potential conflict of interest.

## Publisher’s Note

All claims expressed in this article are solely those of the authors and do not necessarily represent those of their affiliated organizations, or those of the publisher, the editors and the reviewers. Any product that may be evaluated in this article, or claim that may be made by its manufacturer, is not guaranteed or endorsed by the publisher.
